# Dynamic multiscaling in stochastically forced Burgers turbulence

**DOI:** 10.1038/s41598-023-29056-3

**Published:** 2023-05-02

**Authors:** Sadhitro De, Dhrubaditya Mitra, Rahul Pandit

**Affiliations:** 1 Centre for Condensed Matter Theory, Department of Physics, Indian Institute of Science, Bangalore, 560012 India; 2 NORDITA, KTH Royal Institute of Technology and Stockholm University, Roslagstullsbacken 23, 10691 Stockholm, Sweden

**Keywords:** Statistical physics, thermodynamics and nonlinear dynamics, Statistical physics, Physics

## Abstract

We carry out a detailed study of dynamic multiscaling in the turbulent nonequilibrium, but statistically steady, state of the stochastically forced one-dimensional Burgers equation. We introduce the concept of interval collapse time, which we define as the time taken for a spatial interval, demarcated by a pair of Lagrangian tracers, to collapse at a shock. By calculating the dynamic scaling exponents of the moments of various orders of these interval collapse times, we show that (a) there is not one but an infinity of characteristic time scales and (b) the probability distribution function of the interval collapse times is non-Gaussian and has a power-law tail. Our study is based on (a) a theoretical framework that allows us to obtain dynamic-multiscaling exponents analytically, (b) extensive direct numerical simulations, and (c) a careful comparison of the results of (a) and (b). We discuss possible generalizations of our work to higher dimensions, for the stochastically forced Burgers equation, and to other compressible flows that exhibit turbulence with shocks.

## Introduction

Studies of statistically homogeneous and isotropic turbulence, one of the most important examples of a non-equilibrium steady state (NESS), lie at the interfaces between non-equilibrium statistical mechanics, fluid dynamics, and spatiotemporal chaos. In this NESS, the turbulent fluctuations of the flow velocity span wide ranges of spatial and temporal scales; and the correlation or structure functions, which characterize these fluctuations, display power laws in space and time^1–8^. It has been suggested several times that these power-law forms are like those seen in correlation functions in equilibrium critical phenomena^9–13^. Perhaps the simplest example of a power-law form in such turbulence is the scaling of the energy spectrum, $E(k)\sim k^{-\alpha }$, for wave numbers *k* in the *inertial range,* $\eta ^{-1}\ll k \ll L_{\textrm{I}}^{-1}$, where $\eta$ and $L_{\textrm{I}}$ are, respectively, the energy-dissipation and energy-injection length scales^1,14^. The phenomenological theory, proposed by Kolmogogorov in 1941 (K41)^15^, yields the exponent $\alpha ^{K41} = 5/3$, but the intermittency of turbulence leads to multifractal corrections to this and to other exponents (see below and, e.g., Refs.^1,14,16,17^). To understand this multifractality we must go well beyond^1,14,16,17^ the theoretical framework that is used to explain power-law correlation functions at equilibrium critical points^9–11^.

Multifractal fluctuations lead to considerable theoretical challenges when we move from equal-time to time-dependent correlation functions^2–8^. In simple critical phenomena, power-law forms for time-dependent correlation functions come from the divergence of the correlation time, $\tau$, that is related to the diverging correlation length, $\xi$, by the *dynamic scaling Ansatz*^18–20^, $\tau \sim \xi ^\theta$, with $\theta$ the *dynamic scaling exponent*. In turbulence, we can define time scales in a variety of different ways. Although the K41 theory suggests simple dynamic scaling, wherein all time scales are characterized by the same dynamic exponent $\theta ^{K41} =2/3$, direct numerical simulations (DNSs) and heuristic understanding of various models of turbulence suggest *dynamic multiscaling*, i.e., different time scales are linked to spatial scales via different dynamic exponents^4–8,21,22^. L’vov *et al.*^4^ had proposed that the characteristic times of eddy-velocity correlation functions of various orders are linked to the eddy sizes through multiple dynamic exponents, which can be related to the equal-time structure function exponents by linear bridge relations. Thereafter, Mitra and Pandit^5^ not only confirmed this from their shell-model DNS, but also showed that the dynamic exponents depend specifically on how the time scales are defined. Additionally, they showed that simple dynamic scaling is obtained if the velocity field is of the type of that in the Kraichnan model^23,24^; but *bona fide* non-trivial dynamic multiscaling is obtained if the advecting velocity itself exhibits dynamic multiscaling^25^.

Note that, in the context of dynamic multiscaling, passive scalar advection and shell models are relatively simpler to analyze. The governing equation of the former is *linear* in the scalar concentration. In shell models, the velocity modes, ${\hat{u}}(k)$, interact only with those belonging to the nearest- or next-neighbour shells in the the wave number space (see, e.g., Refs.^14,26^). However, in hydrodynamic turbulence, the velocity advection term is nonlinear and it couples all the velocity Fourier modes to one another. Because of this, small eddies (small-*k* modes) are advected by the large eddies (large-*k* modes), whence we get a *sweeping effect*^27^ that yields eddy lifetimes that are linearly proportional to the eddy size, i.e., the dynamic exponent is unity. This sweeping effect, which masks the underlying dynamic multiscaling of turbulent flows, is a manifestation of Taylor’s hypothesis.

For incompressible turbulence, Belinicher and L’vov^28^ had suggested that quasi-Lagrangian (QL) velocities, calculated in the reference frame of a Lagrangian particle or tracer, should be free from sweeping effects. This was shown explicitly by Ray *et al.*^7^, who quantified the dynamic multiscaling of forward-cascade turbulence in the incompressible two-dimensional ($d=2$) Navier–Stokes equation. They also showed that sweeping effects can be suppressed by friction, which removes energy from a flow at all spatial scales. Biferale *et al.*^21^ used QL velocities to obtain dynamic multiscaling for turbulence in the incompressible three-dimensional ($d=3$) Navier–Stokes equation. However, characterization of dynamic multiscaling via the QL approach has not been attempted yet in compressible turbulence, in general, and in Burgers turbulence, in particular.

The Burgers equation is the simplest compressible hydrodynamic partial differential equation which was originally introduced for the study of pressure-less gas dynamics^29,30^. It is often used in cosmology to model the formation and distribution of large-scale structures in the universe^31^, under the *adhesion approximation *^32^. Since it can be derived from the spatial derivative of the Kardar-Parisi-Zhang (KPZ) equation, the noisy Burgers equation also has applications in interface growth and roughening^33,34^. In fluid dynamics, the Burgers equation is is often used as a testing ground for statistical theories of turbulence^35,36^ because it has the same nonlinear term as the Navier–Stokes equation. The *d*-dimensional Burgers equation can be exactly solved via the Hopf-Cole transformation for smooth initial conditions and/or smooth forcing^30,37,38^; in the inviscid limit, this yields a maximum principle for a velocity potential^35,36,39^. On the other hand, Burgers turbulence (sometimes referred to as Burgulence) comprise solutions of the Burgers equation with (a) random initial conditions and/or, (b) stochastic forcing. Earlier DNSs of $d=1$ stochastically forced Burgers turbulence have shown that the velocity structure functions in the NESS display multiscaling^2,3,40–45^. For a Gaussian white-in-time forcing with an amplitude spectrum, ${\hat{f}}(k)\sim k^{-1/2}$, it has been conjectured that this multiscaling could be a numerical artifact^45^ which could wane on increasing the spatial resolution of the DNS, and be replaced by bifractal scaling.

The study of dynamic scaling in the stochastically forced Burgers equation is not as well developed as it is in incompressible fluid turbulence. An earlier DNS-based study of certain time-dependent, Eulerian velocity structure functions of this model yielded a single dynamic exponent of unity^2^ that was attributed to the sweeping effect. We go beyond this Eulerian study and the QL investigations of incompressible fluid turbulence^4–8,21,22^.

In compressible flows, Lagrangian tracers get trapped at shocks because of which the QL transformation might not be adequate for the removal of sweeping effects. For the $d=1$ stochastically forced Burgers turbulence, we overcome this difficulty by using *a pair of tracers* separated initially by a Lagrangian interval of length, $\ell$. We then compute the *interval-collapse time*, $\tau _{\textrm{col}}$, which we define as the time at which this interval collapses to a point at a shock. We find that $\tau _{\textrm{col}}$ depends on both $\ell$ and the location of the interval. Hence we compute, for each value of $\ell$, the probability distribution function (PDF) of $\tau _{\textrm{col}}$ and extract a hierarchy of time scales, $T^{\textrm{p}}_{\textrm{col}}(\ell )$, from its order-*p* moments. We make the dynamic-scaling *Ansätze*, $T^{\textrm{p}}_{\textrm{col}}(\ell )\sim \ell ^{z^{\textrm{p}}_{\textrm{col}}}$, and obtain therefrom the *interval-collapse exponents* $z^{\textrm{p}}_{\textrm{col}}$ for different values of *p*. We find from our high-resolution DNS that $z^{\textrm{p}}_{\textrm{col}}$ is not a linear function of *p*. This indicates dynamic multiscaling. We develop a theory for the *p*-dependence of $z^{\textrm{p}}_{\textrm{col}}$, which indicates that $z^{\textrm{p}}_{\textrm{col}}$ should be a piecewise linear function of *p*. Our theory also yields an analytical expression for the PDF of $\tau _{\textrm{col}}$. The PDF is non-Gaussian and has a power-law tail; we compare this with the results of our DNS. We also show from our DNS that the QL approach is indeed unable to suppress sweeping effects. Furthermore, we examine the extension of our *interval-collapse* framework for examining dynamic scaling in the stochastically forced Burgers equation to dimensions $d > 1$ and compressible Navier–Stokes turbulence, and outline the challenges in carrying out such studies.

The remainder of this paper is organized as follows: In the “Model and numerical methods” section we define the stochastically forced Burgers equation and outline the numerical methods that we use. In the “Interval-collapse times” section, we investigate the statistical properties of the interval-collapse times, $\tau _{\textrm{col}}$, and show how to calculate the interval-collapse exponents $z^{\textrm{p}}_{\textrm{col}}$. In the “Time-dependent structure functions” section, we explore dynamic multiscaling via QL velocity structure functions. Finally, in the “Conclusion and discussions” section, we discuss the significance of our results and propose ways of extending our approach to $d > 1$ and compressible turbulent flows with shocks.

**Figure 1:**
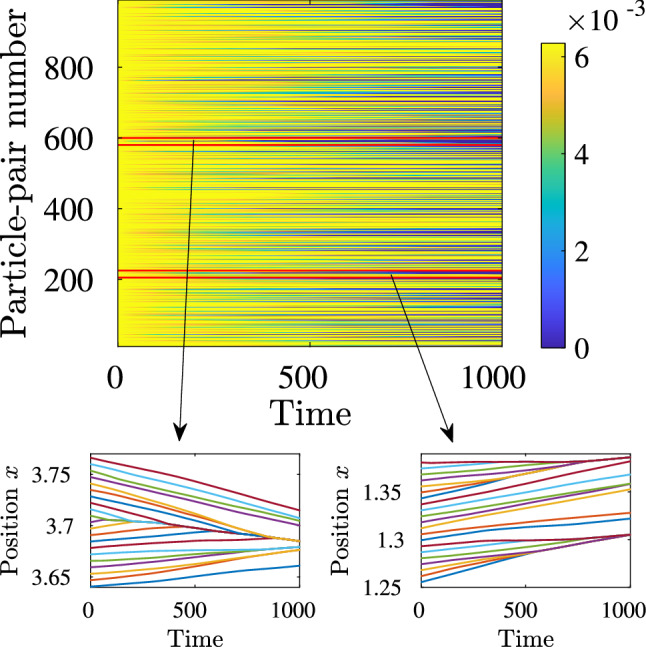
(Color online) Space–time plots of representative tracer trajectories (run R1) for initially equi-spaced tracers; we choose 1000 tracers for ease of visualization here. The vertical axis gives the nearest-neighbor tracer-pair number (the tracers are numbered in ascending order and the pair number *i* denotes two tracers that are initially at $2\pi i/1000$ and $2\pi (i+1)/1000$); the horizontal axis denotes the time (in units of iteration number). The color bar denotes the separation between the tracers within each of the nearest-neighbor tracer pairs. As time progresses, the separation between the tracers in such pairs decreases. Bottom panel: Expanded versions of the tracks of the tracers (distinguished by different colors) enclosed within the red-bordered rectangular boxes in the top panel.

## Model and numerical methods

The $d=1$ stochastically forced Burgers equation that we consider is

1 $$\begin{aligned} \partial _tu+u\partial _xu=\nu \partial _{xx}u+f(x,t), \end{aligned}$$

where *u*(*x*, *t*) is the fluid velocity at position *x* and time *t*, $\nu$ is the kinematic viscosity, and *f* is a zero-mean, Gaussian white-in-time random force whose Fourier components, ${\hat{f}}(k,\,t)$, satisfy

2 $$\begin{aligned} \langle {\hat{f}}(k,\,t){\hat{f}}(k',\,t')\rangle \sim k^{-\beta }\delta (k+k')\delta (t-t'), \end{aligned}$$

where *k* is the the wave number. We choose $\beta =1$, because, at the level of a one-loop RG, this choice of $\beta$ yields a K41-type energy spectrum $E(k) \equiv \langle |{\hat{u}}(k,t)|^2 \rangle \sim k^{-5/3}$, for *k* in the inertial range, where ${\hat{u}}(k,t)$ is the Fourier transform of *u*(*x*, *t*), and the angular brackets denote a time average over the NESS^2,42,43,45^.

In our DNSs of Eqs. (1) and (2), we use periodic boundary conditions in a domain of length $L=2\pi$ and *N* collocation points. To achieve high spatial resolutions we use $N=2^{16}$ (Run R1) and $N=2^{20}$ (Run R2). We employ a standard pseudospectral method with the 2/3-dealiasing rule^46^. For time-stepping we use the implicit Euler-Maruyama scheme^47,48^. We generate the stochastic force, ${\hat{f}}(k,t)$, in Fourier space, with a high-wave-number cutoff at $k_c = N/8$. We have confirmed that our results remain unchanged even if we evolve the unforced equation by using the second-order exponential time-differencing Runge-Kutta method and then add the forcing term, ${\hat{f}}(k,t)\sqrt{\delta t}$, to the velocity field at the end of every time step of step size $\delta t$. The parameters for our DNS runs are given in the Supplementary Material. Most of our DNSs and data analysis have been carried out on a GPU cluster with NVIDIA Tesla K20 accelerators. The energy spectrum, *E*(*k*), in the NESS shows inertial-range scaling over two decades in *k*; a typical snapshot of a steady-state velocity profile and a compensated plot of *E*(*k*), for run R2, are shown in Fig. 1 of the Supplementary Material.

After our system reaches its NESS, we introduce $N_p$ equi-spaced Lagrangian particles (or tracers), whose equations of motion are

3 $$\frac{d}{{dt}}R_{i} (t) = U_{i} \quad \:{\mkern 1mu} {\text{and}}\quad U_{i} = u(x,t)\delta (x - X_{i} ),$$

where $X_i$ and $U_i$ are the instantaneous position and velocity, respectively, of the $i-$th tracer, and $\delta (x)$ is the Dirac delta function. We solve (3) by using the forward-Euler method. The $\delta (x-X_i)$ factor is implemented by linear interpolation (We use a small viscosity, so the shocks are well-resolved, typically spread over 6 collocation points. Hence linear interpolation is sufficient to obtain the Lagrangian velocity of the passive tracers.). As time progresses, the tracers cluster at the shocks, as we show in Fig. 1.

**Figure 2:**
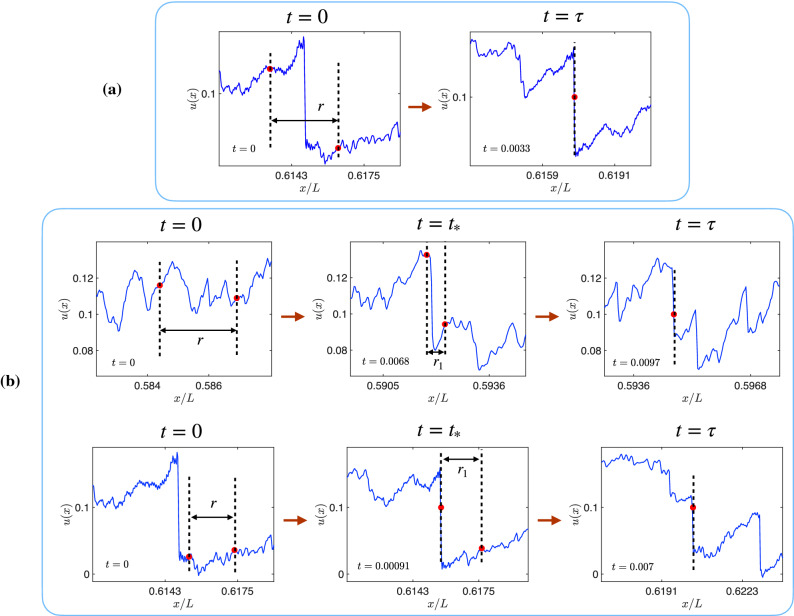
Examples of the possible cases of collapse of an interval of initial length *r* from our DNSs: (**a**) The collapsing interval contains a shock at $t=0$ and it collapses at that shock at $t=\tau$. (**b**) The interval has no shock at $t=0$ and collapses, at $t=\tau$, at a shock which either appears within it (upper row) or merges with one of its ends (lower row) at time $t=t_{*}$. In all figures, *t*, $t_{*}$ and $\tau$ have been non-dimensionalized with $T_{\textrm{L}}$.

## Interval-collapse times

We define $\tau _{\textrm{col}}(\ell )$ as the time taken for an interval, with initial length $\ell$, to collapse to a point at a shock, with $t=0$ the time at which we seed the flow with tracers. We consider many such intervals at $t=0$ and address the following questions:

- *What is the PDF of* $\tau _{\textrm{col}}(\ell )$?
- *How does this PDF depend on* $\ell$?

We start by investigating the moments of this PDF.

**Figure 3:**
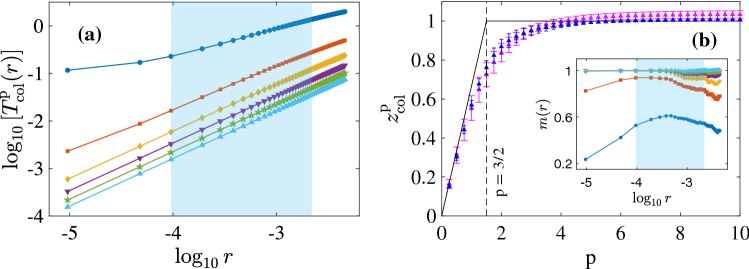
(**a**) Log–log plots of $T^{\textrm{p}}_{\textrm{col}}(r)$ versus *r* for $p=1$ (dark blue circles), $p=2$ (red squares), $p=3$ (yellow diamonds), $p=4$ (violet inverted triangles), $p=5$ (green pentagrams), and $p=6$ (light blue triangles) from run R2; we extract the exponents, $z^{\textrm{p}}_{\textrm{col}}$, by calculating the local slopes of these graphs across the shaded region. (**b**) The exponent, $z^{\textrm{p}}_{\textrm{col}}$, versus *p* from runs R1 (pink triangles) and R2 (blue triangles). The solid line shows our theoretical prediction, Eq. (17b). The vertical dashed line marks the kink in the predicted curve which occurs at $p=3/2$. *Inset:* The local slope, *m*(*r*), as a function of *r*, the color and symbol coding being the same as that in (**a**). The means and standard deviations of *m*(*r*), calculated over the shaded region, yield the exponents, $z^{\textrm{p}}_{\textrm{col}}$, and their error bars, respectively.

### Dynamic scaling: theoretical considerations

We extract a hierarchy of time scales, $T^{\textrm{p}}_{\textrm{col}}$, by normalizing order-*p* moments of the PDF of $\tau _{\textrm{col}}$ by the the large-eddy turnover time, $T_{\textrm{L}}=L_{\textrm{I}}/u_{\textrm{rms}}$, with $u_{\textrm{rms}}$ the root-mean-square velocity, as follows:

4 $$T_{{{\text{col}}}}^{{\text{p}}} (r) \equiv \frac{1}{{T_{{\text{L}}}^{{p - 1}} }}\left\langle {\tau _{{{\text{col}}}} (r,x)^{p} } \right\rangle _{x} ;$$

$\langle \cdot \rangle_x$ denotes an average over different spatial locations *x* and $r\equiv \ell /L$ is the non-dimensionalized interval length. The velocity difference, $\delta u(r)$, across inertial range separations $\eta /L\ll r\ll 1$ in a turbulent flow scales as $\delta u(r)\sim v_0r^h$, where $v_0 \equiv u_0/u_{\textrm{rms}}$, the large-scale fluctuating velocity field is $u_0$, and *h* is the scaling exponent of the velocity fluctuations. Theoretical considerations suggest^45^ that the NESS of Eqs. (1) and (2) exhibits the following bifractal scaling, for equal-time velocity structure functions:

5a $$\begin{aligned} \delta u(r,x)&\equiv u(x+r) - u(x) \, ; \end{aligned}$$

5b $$\begin{aligned} S_{\textrm{p}}(r)&\equiv \langle \left| \delta u(r,x)\right| ^p\rangle_x \sim r^{\zeta _{p}} \, ; \end{aligned}$$

5c $$\begin{aligned} \zeta _{p}&= {\left\{ \begin{array}{ll} p/3 &{}\text {for}\quad p \le 3 \, ; \\ 1 &{}\text {for}\quad p > 3 \, . \end{array}\right. } \end{aligned}$$

This bifractal scaling is obtained as follows: For an interval of length *r*, (a) $\delta u(r,x) \sim v_0r^h$, if the interval does not contain any shock; (b) $\delta u(r,x) \sim v_0$, independent of *r*, if it does contain a shock. The value of the exponent *h* depends on $\beta$; for $\beta =1$, $h=1/3$. For $r\ll 1$ (More specifically, scaling theories of inviscid turbulent flows are formulated in the limit of $r\rightarrow 0$. However, the order of taking limits must be kept in mind here. First, one should consider the limit $\nu \rightarrow 0$, which sets $\eta \rightarrow 0$, and thereafter take the limit $r\rightarrow 0$.), an interval of length *r* can have at most one shock with a probability $p_r \propto r$ (this holds unless the shocks are distributed on a fractal set, which is not the case here). If we substitute these expressions for $\delta u(r,x)$ in $S_{\textrm{p}}(r)$, we obtain the result for $\zeta _{p}$ in Eq. (5). The DNS results of Ref.^45^ yield multiscaling; but it has been suggested in this study that such multiscaling might be an artifact that should be replaced by the bifractal scaling [Eq. (5)] in the limit of infinite spatial resolution.

We generalize these theoretical arguments to obtain exact expressions for the dynamic scaling exponents of the NESS of Eqs. (1) and (2). At any instant of time *t*, consider two Lagrangian particles at the two ends, *x* and $x+y$, of an interval of length *y*. Then

6 $$\begin{aligned} {\dot{y}}(x) = u(x+y) - u(x) \equiv \delta u(y,x), \end{aligned}$$

where ${\dot{y}}=dy/dt$. The time of collapse of an interval $\tau (r,x)\equiv \tau _{\textrm{col}}(r,x)/T_L$ is obtained by integrating Eq.(6):

7 $$\begin{aligned} \tau (r,x) \sim \int _r^0\frac{dy}{\delta u(y,x)}. \end{aligned}$$

In the small-*r* limit, we have the two cases *A* and *B*, which we describe below and illustrate in Fig. 2.

***(A)*** There is a shock within the collapsing interval of size *r* at $t=0$ (see panel (a) in Fig. 2). In this case $\delta u(y,x) \sim v_0$ is independent of *y*, for all $y\in [0,r]$. Hence, from Eq. (7) we obtain

8 $$\begin{aligned} \tau (r,x) \sim \frac{r}{v_0} . \end{aligned}$$

The probability that an interval of size *r* contains a shock is proportional to *r* (this probability is *ar*, with *a* a constant that does not affect the power laws that we obtain below), so

9 $$\begin{aligned} T^{\textrm{p}}_{\textrm{col},A}(r) \sim r \left( \frac{r}{v_0}\right) ^p \sim r^{p+1}; \quad \text {case A} . \end{aligned}$$

***(B)*** There is no shock in the interval at $t=0$ (see panel (b) in Fig. 2). As we have noted below Eq.(5), $\delta u(r,x) \sim r^h$ for this case. However, either a shock forms within the collapsing interval (first row in panel (b) of Fig. 2) or one of the ends of the interval gets trapped at a shock (second row in panel (b) of Fig. 2), at some time $t_{*}$. The probability $p_{s1}$ of finding an interval of initial size *r*, which collapses as in the first row of panel (b) in Fig. 2, is $p_{s1}\equiv br$, where *b* is a constant that does not affect our results for exponents. Similarly, the probability of finding an interval, which collapses as in the second row of panel (b) in Fig. 2, is $p_{s2} \equiv 1-(a+b)r$. We must now distinguish between the following two sub-cases.

*(B1)* $t_{*}$ is very close to $\tau (r,x)$, i.e., $t_{*}\sim \tau (r,x)$. Furthermore, $\delta u(y,x) \sim y^h$, for all $y\in [\eta ,r]$, where $\eta \rightarrow 0$ in the inviscid limit; hence, by using Eq. (7), we obtain

10 $$\begin{aligned} \tau (r,x) \sim \frac{1}{v_0}r^{(1-h)}. \end{aligned}$$

Hence, the contribution from this case to $T^{\textrm{p}}_{\textrm{col}}(r)$ is

11 $$\begin{aligned}{} & {} T^{\textrm{p}}_{\textrm{col},B1}(r) \sim w_1\tau ^p p_{s1} + w_2\tau ^p p_{s2}\nonumber \\{} & {} \quad \sim w_2 r^{p(1-h)} + {\mathcal {O}}(r^{p(1-h)+1}); \quad \text {case B1}. \end{aligned}$$

$w_1$ and $w_2$ are the weights associated with the respective types of interval collapse (see above). The first term on the right-hand-side (RHS)of (11) is the dominant one in the small-*r* limit.

*(B2)* $t_{*}$ is significantly smaller than $\tau$, i.e., $t_{*}<\tau$, so case *(A)* follows after the time $t_{*}$, i.e.,

12 $$\begin{aligned} \tau (r,x) \sim t_{*}+ \frac{r_1}{v_0} , \end{aligned}$$

where $r_1$ is the size of the interval when the shock forms. Then, by integrating Eq. (7) from $t=0$ to $t_{*}$, during which time $\delta u(y,x) \sim y^h$, we obtain

13 $$\begin{aligned} v_0t_{*}\sim \frac{1}{1-h}\left[ r_1^{1-h} - r^{1-h} \right] . \end{aligned}$$

By solving for $r_1$ from Eq. (13), substituting it in Eq. (12), expanding in powers of *r*, and, in the small-*r* limit, neglecting terms of order $r^h$ compared to unity, we obtain

14 $$\begin{aligned} \tau (r,x) \sim t_{*}+ \frac{r}{v_0}. \end{aligned}$$

Hence,

15 $$\begin{aligned} T^{\textrm{p}}_{\textrm{col},B2}(r) \sim w_3r + w_4r^p; \quad \text {case B2}. \end{aligned}$$

Here, we have incorporated the contributions from the two modes of interval collapse (cf. Eq. (11)), expanded the *p*-th power of $\tau$ from Eq. (14), and kept only the leading-order terms for $r\ll 1$; $w_3$ and $w_4$ are the weights of the respective leading-order contributions.

We combine the contributions from cases (*A*), (*B*1), and (*B*2) [Eqs. (9), (11), and (15) respectively] to obtain

16 $$\begin{aligned} T^{\textrm{p}}_{\textrm{col}}(r) \sim W_1 r^{p+1} + W_2 r^{p(1-h)} + W_3 r + W_4 r^p, \end{aligned}$$

where $W_1$, $W_2$, $W_3$ and $W_4$ are the respective weights of these contributions. For $r \ll 1$ we can ignore the first term, on the right-hand-side (RHS) of (16), compared to the third and the fourth term compared to the second. Further, for $0 < p\le 1/(1-h)$, the second term dominates over the third, otherwise, the third term dominates. Consequently, the dynamic-scaling exponents, defined by

17a $$\begin{aligned} T^{\textrm{p}}_{\textrm{col}}(r)&\sim r^{z^{\textrm{p}}_{\textrm{col}}} \,,\quad \text {are} \end{aligned}$$

17b $$\begin{aligned} z^{\textrm{p}}_{\textrm{col}}&= {\left\{ \begin{array}{ll} \frac{2p}{3} &{}\text {for}\quad 0 \le p\le \frac{3}{2} \, ,\\ 1 &{}\text {for}\quad p > \frac{3}{2} \, . \end{array}\right. } \end{aligned}$$

In (17b), we have set $h=1/3$. Thus, the bifractal scaling of equal-time velocity structure functions, (5), is accompanied by *dynamic multiscaling*, since it is possible to obtain infinitely many time scales, $T^{\textrm{p}}_{\textrm{col}}(r)$, with each having a different scaling exponent, $z^{\textrm{p}}_{\textrm{col}}$.

### Dynamic scaling: direct numerical simulations

We now use data from our DNSs to calculate $T^{\textrm{p}}_{\textrm{col}}(r)$; and we present log–log plots of it versus *r*, for $1 \le p \le 6$, in Fig. 3a. For all these values of *p*, $T^{\textrm{p}}_{\textrm{col}}(r)$ shows power-law scaling over almost a decade and a half in *r*. From these power-law regions [in the blue-shaded rectangle in Fig. 3a], we extract the interval-collapse exponents $z^{\textrm{p}}_{\textrm{col}}$ via a local-slope analysis of the curves. In Fig. 3b, we plot $z^{\textrm{p}}_{\textrm{col}}$ as a function of *p*, with pink and blue triangles for $z^{\textrm{p}}_{\textrm{col}}$ from runs R1 and R2, respectively. In the inset of Fig. 3b, we plot the local slopes, *m*(*r*), whose means and standard deviations across the blue-shaded portion of the inset yield $z^{\textrm{p}}_{\textrm{col}}$ and its error bars, respectively. The black lines indicate the predictions of Eq. (17b). Note that the values of $z^{\textrm{p}}_{\textrm{col}}$ from runs R1 and R2 lie within error bars of each other; moreover, the exponents from the higher-resolution run R2 lie slightly closer to the theoretical prediction than those from run R1. The deviation from this prediction is most pronounced near the transition value, $p=3/2$.

Therefore, our numerical results for $z^{\textrm{p}}_{\textrm{col}}$ [Fig. 3b] indicate *dynamic multiscaling*. We remark that the small deviation of our DNS results for $z^{\textrm{p}}_{\textrm{col}}$ from its predicted values [Eq. (17b)] are similar to what has been observed^45^ for the equal-time velocity-structure-function exponents $\zeta _{p}$. It is possible that this small deviation could decrease as we increase the resolution of our DNS. However, given the tiny difference between the results for runs R1 and R2, we might well have to go to DNS resolutions that are computationally prohibitive before our results for $z^{\textrm{p}}_{\textrm{col}}$ approach the result of Eq. (17b). Note that our DNS run R2 is, to date, the highest-resolution DNS of Eqs. (1) and (2).

**Figure 4:**
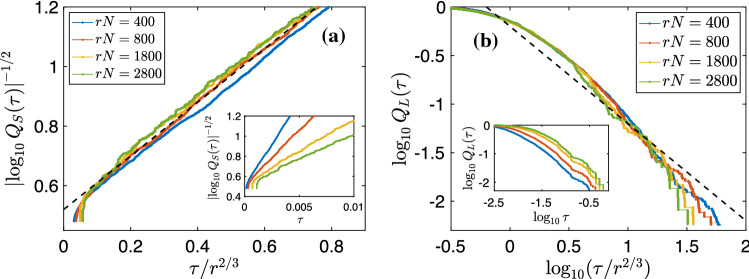
Plots for run R2: (**a**) $\left| \log _{10}Q_S(\tau )\right| ^{-1/2}$ versus $\tau /r^{2/3}$ for different values of *rN*; the black dashed straight line represents Eq. (26). Inset: $\left| \log _{10}Q_S(\tau )\right| ^{-1/2}$ versus $\tau$ for different values of *rN*. (**b**) Log–log plots of the c-CPDFs, $Q_L$, versus $\tau /r^{2/3}$ for different values of *rN*; the black dashed line has a slope of $-1$. The c-CPDFs collapse, reasonably well, onto one curve when we scale $\tau$ by $r^{2/3}$; this curve exhibits a power-law decay with an exponent that deviates slightly from the prediction of $-1$ [see Eq. (27)]. *Inset:* Log–log plots of $Q_L$ versus $\tau$ for different values of *rN*.

### Probability distribution functions (PDFs) of interval-collapse times

We turn now to the PDFs, $\Phi (\tau _{\textrm{col}})$, of $\tau _{\textrm{col}}$ for different values of *r*; for notational convenience we suppress the argument, *r,* of $\tau \equiv \tau _{\textrm{col}}/T_L$ in this subsection.

#### PDFs: theoretical considerations

We use the relation

18 $$\begin{aligned} \Phi (\tau )={\mathcal {P}}(v_0)\left| \frac{dv_0}{d\tau }\right| , \end{aligned}$$

where ${\mathcal {P}}(v_0)$ is the PDF of $v_0$; for the Burgers equation with an external force that is limited to small *k*, this PDF is known to be a Gaussian^49^. In our DNSs of Eqs. (1) and (2), we find this PDF to be a Gaussian too (see Fig. 2 in the Supplementary Material), i.e.,

19 $$\begin{aligned} {\mathcal {P}}(v_0)\sim e^{-v_0^2/2}. \end{aligned}$$

We now examine the forms of $\Phi (\tau )$ for the three cases *(A)*, *(B1)*, and *(B2)* that we have considered in the “Dynamic scaling: theoretical considerations” section.

***(A)*** There is a shock in the interval of size *r* at $t=0$. The probability of finding such an interval is $p_r \propto r$. From Eq. (8), $v_0\sim r/\tau$. Hence, for a given value of $r\ll 1$,

20 $$\begin{aligned} \Phi _A(\tau ) = {\mathcal {P}}(v_0)\left| \frac{dv_0}{d\tau }\right| p_r \sim \frac{r^2}{\tau ^2}\exp \left( -\frac{r^2}{2\tau ^2}\right) . \end{aligned}$$

***(B)*** There are no shocks within the interval at $t=0$, as in the “Dynamic scaling: theoretical considerations” section. The interval can collapse in one of the two ways illustrated in panel (b) of Fig. 2 and can occur with probability $p_{s1}\equiv br$ or $p_{s2}\equiv 1-(a+b)r$, depending on its mode of collapse (see above). We consider the following sub-cases:

*(B1)* $t_{*}\sim \tau$ and $v_0\sim r^{1-h}/\tau$, so, to leading order in *r*, for $r\ll 1$,

21 $$\begin{aligned} \Phi _{B1}(\tau ) \sim \frac{r^{1-h}}{\tau ^2}\exp \left( -\frac{r^{2(1-h)}}{2\tau ^2}\right) . \end{aligned}$$

*(B2)* Here, $t_{*}<\tau$ and $v_0\sim r/(\tau -t_{*})$, hence, to leading order in *r*, for $r\ll 1$,

22 $$\begin{aligned}{} & {} \Phi _{B2}(\tau ) \sim \sum _{t_{*}}\left[ \frac{r}{(\tau -t_{*})^2}\exp \left\{ -\frac{r^2}{2(\tau -t_{*})^2}\right\} \right] \Theta (\tau -t_{*})w(t_{*}), \end{aligned}$$

where the summation is over all possible values of $t_{*}$, with weights $w(t_{*})$, and $\Theta$, the Heaviside step function, ensures that, for a given value of $t_{*}$, the contributions for all $\tau \le t_{*}$ is zero.

By combining Eqs. (20)-(22) and substituting $h=1/3$, we get

23 $$\begin{aligned}{} & {} \Phi (\tau )\sim \mu _1\frac{r^{2/3}}{\tau ^2}\exp \left( -\frac{r^{4/3}}{2\tau ^2}\right) +\mu _2\frac{r^2}{\tau ^2}\exp \left( -\frac{r^2}{2\tau ^2}\right) + \mu _3 \Phi _{B2}(\tau ) , \end{aligned}$$

where $\mu _1$, $\mu _2$, and $\mu _3$ are the weights of three contributions to $\Phi (\tau )$. We can recover the values $z^{\textrm{p}}_{\textrm{col}}$, given in Eq. (17b), by calculating the moments of $\Phi (\tau )$ (see the Supplementary Material for detail), and thus check our derivation of $\Phi (\tau )$. In summary, the contributions to the order-*p* moments of $\Phi (\tau )$, from the first two terms on the RHS of Eq. (23), scale as $r^{2p/3}$ and $r^{p+1}$, respectively. The leading-order contribution from the last term, $\Phi _{B2}$, scales linearly with *r* and this comes from the binomial expansion of the term $\tau ^p$ after making the substitution, $s=r/(\tau -t_{*})$. Note that Eq. (23) explicitly demonstrates that there is no unique dynamic scaling exponent; the arguments of the two exponentials suggest dynamic exponents of 2/3 and 1, respectively; whereas, the power-law factors multiplying the exponentials suggest dynamic exponents of 1/3, 1 and 1/2, respectively. Furthermore, this PDF is not a Gaussian.

#### PDFs: direct numerical simulations (DNSs)

We now compare the asymptotic properties of $\Phi (\tau )$ [Eq. (23)] with the results that we obtain from our DNSs for run R2. Since insufficient sampling of rare events in the two tails of $\Phi (\tau )$ can cause inaccuracies in the calculation, we instead investigate the tails of the cumulative PDF (CPDF) $Q_S(\tau )$, and the complementary CPDF (c-CPDF), $Q_L(\tau )$. $Q_S$ and $Q_L$ are defined as follows:

24 $$\begin{aligned} Q_S(\tau )=\int _{0}^{\tau } \Phi (\tau ')d\tau ' ; \quad Q_L(\tau )=\int _{\tau }^{\infty } \Phi (\tau ')d\tau '. \end{aligned}$$

We calculate $Q_S$ and $Q_L$ from our data by using the rank-order method, since it circumvents binning errors.

In the $\tau \rightarrow 0$ limit, the contribution from $\Phi _{B2}(\tau )$ is negligible. So, for fixed $r \ll 1$, $Q_S(\tau )$ scales as

25 $$\begin{aligned} Q_S(\tau )\sim \textrm{erfc}\left( \frac{r^{2/3}}{\tau }\right) \sim \frac{\tau }{r^{2/3}}\exp \left( -\frac{r^{4/3}}{\tau ^2}\right) , \end{aligned}$$

in this limit, where $\textrm{erfc}(x)$ is the complementary error function. To derive the second step of Eq. (25), we use the leading-order asymptotic form of $\textrm{erfc}(x)$ in the limit of $x\rightarrow \infty$. Furthermore, $\frac{r^{4/3}}{\tau ^2}\gg \left| \log \left( \frac{\tau }{r^{2/3}}\right) \right|$, so, in the limit of $\tau \rightarrow 0$, we get

26 $$\begin{aligned} \Big |\log Q_S(\tau )\Big |\sim \left( \frac{\tau }{r^{2/3}}\right) ^{-2}. \end{aligned}$$

In Fig. 4a, we plot $\left| \log _{10}Q_S\right| ^{-1/2}$ versus $\tau /r^{2/3}$ for different values of *rN*, with *N* the number of collocation points. In the inset, we plot $\left| \log _{10}Q_S\right| ^{-1/2}$ versus $\tau$ for different values of *rN*. The plots in Fig. 4a collapse, fairly well, onto the dashed straight line [Eq. (26)], thereby providing numerical support for our $\tau \rightarrow 0$ result for $Q_S(\tau )$.

For large-$\tau$, $\tau \gg r^{2/3}$, we expand the RHS of (23) in a Taylor series, retain only the leading-order contribution for $r\ll 1$, and arrive at the following form for the c-CPDF:

27 $$\begin{aligned} Q_L(\tau ) \sim \left( \frac{\tau }{r^{2/3}}\right) ^{-1}. \end{aligned}$$

In Fig. 4b, we present log–log plots of $Q_L(\tau )$ versus $\tau /r^{2/3}$ for different values of *rN*; the inset shows log–log plots of $Q_L(\tau )$ versus $\tau$. We observe a reasonable collapse of the c-CPDFS onto a curve, which is close to, but steeper than, the straight dashed line that represents Eq. (27). This discrepancy arises because of the higher-order terms, which we have neglected in the Taylor expansion of the RHS of Eq. (23), and which have the same sign as that of the dominant term, so we expect the curves of $Q_L$ to be steeper for moderately large values of $\tau /r^{2/3}$. The corrections because of these higher-order terms reduce on increasing $\tau$ or decreasing *r*. Consequently, in Fig. 4a, we observe that, for small values of *r*, the plots of $Q_L(\tau )$ (blue and red curves) tend to align with the dashed black line with slope $-1$, at large values of $\tau /r^{2/3}$; this alignment is not so good as *r* increases.

**Figure 5:**
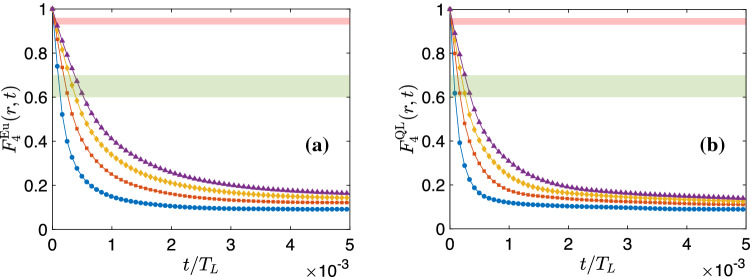
Plots of (**a**) $F^{\textrm{Eu}}_{\textrm{p}}(r,t)$ and (**b**) $F^{\textrm{QL}}_{\textrm{p}}(r,t)$ versus *t* for $p=4$ and $rN=100$ (blue circles), 200 (red squares), 300 (yellow diamonds) and 400 (violet triangles). In both figures, the red and green shaded areas demarcate the regimes $0.93<F_{\textrm{p}}(r,t)<0.96$ and $0.6<F_{\textrm{p}}(r,t)<0.7$, respectively. Within each of these regimes, the integral-time-scale exponents remain unchanged (within numerical error bars).

**Figure 6:**
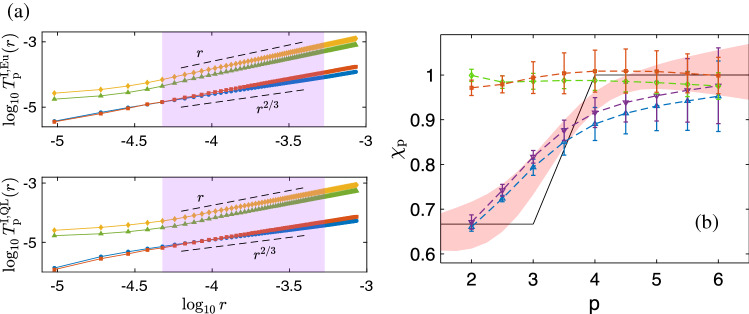
Plots for run R2: (**a**) Log–log plots of the integral time scales, $T^{\mathrm{I,\,Eu}}_{\textrm{p}}(r)$ and $T^{\mathrm{I,\,QL}}_{\textrm{p}}(r)$, versus *r*. In both panels, the blue circles and red squares correspond to orders $p=2$ and $p=3$, respectively, with $\lambda =0.95$ (see text); points obtained with $\lambda =0.65$ are indicted by yellow diamonds and green triangles for orders $p=2$ and $p=3$, respectively. The dashed lines denote the scaling forms $r^{2/3}$ (predicted from (31) for $p=2$ and $p=3$) and $r^1$. The violet shaded area indicates the regime in which we carry out local-slope analyses. (**b**) Blue and violet triangles are the integral-time-scale exponents, $\chi ^{I,\,QL}_p$ and $\chi ^{I,\,Eu}_p$, respectively, calculated with $\lambda =0.95$; the shaded area is the QL-bridge-relation prediction obtained by substituting $\zeta ^{\textrm{QL}}_{\textrm{p}}$ (see Fig. 3 in the Supplementary Material) in Eq. (30); the black lines are the predictions from Eq. (31); the red squares and green diamonds represent $\chi ^{I,\,QL}_p$ and $\chi ^{I,\,Eu}_p$, respectively, calculated with $\lambda =0.65$. The Eulerian and QL bridge-relation predictions are equal up to three decimal places.

## Time-dependent structure functions

Studies of dynamic scaling in incompressible-fluid turbulence use time-dependent structure functions^4–7,21,22,50^. We define the order-*p* time-dependent structure function as

28 $$F_{{\text{p}}} (r,t) \equiv \frac{1}{{S_{{\text{p}}} (r)}}\left\langle {\delta u(x,r,t_{0} )[\delta u(x,r,t_{0} + t)]^{{p - 1}} } \right\rangle _{x} ,{\text{ }}$$

where $\delta u(x,r,t)=\left| u(x+r,t)-u(x,t)\right|$. By definition, $F_{\textrm{p}}(r,0)=1$ for all *r*. From $F_{\textrm{p}}(r,t)$ we extract the integral time scales of order *p* and degree 1, as follows:

29 $$\begin{aligned} T^{\textrm{I}}_{\textrm{p}}(r)\equiv \int _{}^{}F_{\textrm{p}}(r,t)dt \sim r^{\chi ^{\textrm{I}}_{p}}, \end{aligned}$$

where $\chi ^{\textrm{I}}_{p}$ is defined as the order-*p* integral time scale exponent. The multifractal model of turbulence allows us to derive the following bridge relations^5–7^ between $\chi ^{\textrm{I}}_{p}$ and the equal-time exponents, $\zeta _{\textrm{p}}$:

30 $$\begin{aligned} \chi ^{\textrm{I}}_{p}=1+\zeta _{\mathrm{p-1}}-\zeta _{\textrm{p}}. \end{aligned}$$

Using Equation.   (5c), we obtain,

31 $$\begin{aligned} \chi ^{\textrm{I}}_{p}= {\left\{ \begin{array}{ll} \frac{2}{3} &{}\text {for}\quad p < 3; \\ \frac{p-1}{3} &{}\text {for}\quad 3\le p\le 4; \\ 1 &{}\text {for}\quad p > 4. \end{array}\right. } \end{aligned}$$

for the $d=1$ Burgers turbulence.

We first calculate the order-*p* Eulerian equal-time and time-dependent structure functions, $S^{\textrm{Eu}}_{\textrm{p}}(r)$ and $F^{\textrm{Eu}}_{\textrm{p}}(r,t)$, respectively, and plot $F^{\textrm{Eu}}_{\textrm{p}}(r,t)$ for $p=4$ in Fig. 5a. We define a time-scale, $\tau _{\textrm{up}}(r)$, such that $F^{\textrm{Eu}}_{\textrm{p}}(r,\tau _{\textrm{up}})=\lambda$. Then, we evaluate Eulerian integral time scales of order *p*, $T^{\mathrm{I,\,Eu}}_{\textrm{p}}(r)$, by integrating $F^{\textrm{Eu}}_{\textrm{p}}(r,t)$ from $t=0$ to $t=\tau _{\textrm{up}}$ [see Equation.   (29)]. We do this for two different regimes of $\lambda$, $\lambda \in [0.93,0.96]$ (large-$\lambda$) and $\lambda \in [0.6,0.7]$ (small-$\lambda$), demarcated by the pink and green regions, respectively, in Fig. 5a. We plot $T^{\mathrm{I,\,Eu}}_{\textrm{p}}(r)$ for these two regimes in the upper panel of Fig. 6a, for $p=2$ and $p=3$. In both regimes, $T^{\mathrm{I,\,Eu}}_{\textrm{p}}(r)$ scale as power-laws within the inertial range. Using (29), we obtain the Eulerian integral time scale exponents, $\chi ^{\mathrm{I,\,Eu}}_{p}$, which we display in Fig. 6b with violet triangles and green diamonds for the large-$\lambda$ and small-$\lambda$ regimes, respectively. While the former set of exponents capture multiscaling and agree with their bridge relation predictions (30), derived from the equal-time Eulerian structure function exponents, $\zeta ^{\textrm{Eu}}_{\textrm{p}}$, (see Fig. 3 in the Supplementary Material) and illustrated by the pink shaded area in Fig. 6, all the exponents in the latter set are unity. In other words, at short times we observe dynamic multiscaling while at later times, we get simple dynamic scaling with an exponent of unity—because of the dominance of sweeping effects at later times. In any turbulent flow, as in ours, small eddies get advected by the large ones. At small times, this effect is not too pronounced if the advecting velocity field is small. Since larger eddies have longer correlation times, the effects of advection become significant at moderate time scales. This leads to a rapid decorrelation of inertial range eddies, in the frame of reference of an Eulerian observer, as a result of which their lifetimes scale linearly with their characteristic lengths. Similar results were obtained earlier by Hayot and Jayaprakash^2^, where they analyzed a different kind of Eulerian structure functions.

In incompressible turbulence, the QL transformation has been successfully used to suppress sweeping effects and uncover the dynamic multiscaling properties^7,21^. We now test its applicability to Burgers turbulence. Following Ref.^28^, we define the QL velocities as

32 $$\begin{aligned} V(x,t)=u(x+R(t),t), \end{aligned}$$

where *R*(*t*) is instantaneous displacement of a Lagrangian particle which was at $x_0$ at $t=0$ (see the Supplementary Material for details). Using *V*(*x*, *t*), we perform exactly the same calculations that we outlined above, the results of which we display in Fig. 5b [QL time-dependent structure function, $F^{\textrm{QL}}_{\textrm{p}}(r,t)$], Fig. 6a [QL integral time scale, $T^{\mathrm{I,\,QL}}_{\textrm{p}}(r)$] and, Fig. 6b [QL integral time scale exponents, $\chi ^{\mathrm{I,\,QL}}_{p}$]. Most importantly and interestingly, we find that the values of $\chi ^{\mathrm{I,\,QL}}_{p}$ match with those of $\chi ^{\mathrm{I,\,Eu}}_{p}$ in both the small-$\lambda$ and large-$\lambda$ regimes. Note that, for a given *p*, both $\chi ^{\mathrm{I,\,Eu}}_{p}$ and $\chi ^{\mathrm{I,\,QL}}_{p}$ remain unchanged, within numerical errorbars, for all values of $\lambda$ within each regime.

Therefore, we conclude that the QL structure functions, which eliminate sweeping effects in incompressible turbulence^4,7,22^, fail to do so in the present case. The qualitative reason for this is that tracers get trapped in shocks and, thereafter, they move with the shock. We conjecture that this inability of QL structure functions to remove sweeping effects holds in all models of compressible turbulence, once shocks are formed. Note that, at short times, $\chi ^{\mathrm{I,\,QL}}_{p}$ agrees with its bridge relation prediction from QL equal-time structure function exponents, $\zeta ^{\textrm{QL}}_{\textrm{p}}$ [$\zeta ^{\textrm{QL}}_{\textrm{p}}$ and $\zeta ^{\textrm{Eu}}_{\textrm{p}}$ are equal up to three places of decimal (see Fig. 3 in the Supplementary Material)]. Note further that, an attempt to derive the bridge relation for collapse–time exponents using the multifractal model for the $d=1$ Burgers turbulence—see the Supplementary Material for details—also fails because we know of no obvious way to incorporate shocks into the multifractal model.

## Conclusion and discussions

We have carried out a detailed study of the dynamics of the stochastically forced Burgers equation [Eqs. (1) and (2)] in one spatial dimension. We have introduced the concept of interval collapse time and have shown that the probability disbrituion of this time is non-Gaussian. Consequently, there is not one but an infinite number of time-scales, associated with a single length scale—we obtain dynamic multiscaling. We have proposed a theoretical framework to calculate the multitude of dynamic exponents. To the best of our knowledge, *this is the first time that such dynamic multiscaling have been analytically elucidated for turbulence in any nonlinear hydrodynamical partial differential equation.* These results are validated by our high-resolution direct numerical simulations. Furthermore, we have explicitly shown that the quasi-Lagrangian methods^4–7,21,22^, which have been used to study dynamic multiscaling in incompressible turbulence, are not suitable for Burgers turbulence because it is compressible.

The natural generalization of the concept of interval collapse time of Lagrangian intervals to compressible turbulence in higher spatial dimensions, i.e., for $d=2$ and $d=3$, is the collapse times of Lagrangian $d-$simplices, i.e., triangles and tetrahedra in $d=2$ and $d=3$, respectively, We have carried out DNSs of the $d=2$ stochastically forced Burgers equation in which case the collapse of a triangle to one with vanishing area can happen in the following ways:

1. all three vertices collapse to a point;
2. any two vertices come together;
3. all three vertices become colinear, with none of them overlapping.

We find that each of these gives different dynamic exponents. The study of the collapse of Lagrangian tetrahedra in $d=3$ requires further care. Their elucidation requires massive high-resolution DNSs which are in progress and will be reported elsewhere.

Compressible turbulent flows in $d=2$ and 3 with shocks appear in the many engineering (e.g., combustion systems and supersonic jets) and astrophyiscal applications (e.g., the stellar wind, interstellar medium, and molecular clouds^51–63^). The generalization of the interval–collapse time to such cases also brings a fresh perspective to the dynamics of these problems. More specifically, it will help us to understand the dynamics of processes that require the agglomeration of masses or chemical species advected by the compressible turbulent flows. The velocity field in such cases can be Helmholtz-decomposed into a solenoidal and a dilatational part^64^. We may attribute the collapse of *d*-simplices to be driven solely by the dilatational component of the flow velocity. Furthermore, the simulations of compressible turbulence in one dimension^65^ obtain the same bifractal scaling for equal-time structure functions as in $d=1$ (stochastically forced) Burgers turbulence. Hence we expect that the study of the collapse of *d*-simplices in Burgers turbulence may give vital clues towards qualitatively understanding the same phenomenon in more realistic compressible flows. Although we expect quantitative differences because, e.g, the statistics of divergence of velocity may be different for Burgers and compressible Navier–Stokes.

Heavy inertial particles in $d=3$ incompressible turbulence also shows clustering—a phenomena that plays a crucial role in many geophysical and astrophysical phenomena. For these cases, it is straightforward to generalize the concept of collapse times to collapse of *d*-simplices whose vertices are occupied by inertial particles. Even in weakly compressible turbulence, clustering of inertial particles influences the rate at which heterogeneous combustion progresses^66,67^. Clustering because of shocks^68^ may have even larger effects.

It would also be fascinating to explore, in detail, the relation of our work with statistical-mechanical studies, carried out in the context of the KPZ equation, which investigate the behavior of passive random walkers following the wrinkles of a rough surface whose fluctuations are determined by this equation^69–72^.

## Supplementary Information

Supplementary Information.

## Data Availability

The codes used to generate and analyze the data in the current study are available from the corresponding author on reasonable request.
