# Supplementary material for: Dynamic multiscaling in stochastically forced Burgers turbulence
Source: Sci Rep. 2023 May 2;13:7151. doi: 10.1038/s41598-023-29056-3 (PMC10154400; doi:10.1038/s41598-023-29056-3)
Supplement: Supplementary file 1 — Supplementary Information. [file 41598_2023_29056_MOESM1_ESM.pdf]

Supplementary Material: Dynamic multiscaling in stochastically forced Burgers turbulence

Sadhitro De,[1,*] Dhrubaditya Mitra,[2,†] and Rahul Pandit[1,‡]

[1] *Centre for Condensed Matter Theory, Department of Physics, Indian Institute of Science, Bangalore 560012, India*

[2] *NORDITA, KTH Royal Institute of Technology and Stockholm University, Roslagstullsbacken 23, 10691 Stockholm, Sweden*

I. NUMERICAL METHODS

Run	N	ν	δt	$L_{\rm I}$	$u_{\rm rms}$	Re	η	T_L	τ_η	$k_{\rm max}\eta$	N_p
R1	2^{16}	10^{-6}	5×10^{-5}	0.87	0.10	8.7×10^{4}	1.44×10^{-4}	8.70	2.1×10^{-2}	3.14	2^{16}
R2	2^{20}	10^{-7}	2×10^{-6}	0.85	0.22	1.6×10^{6}	3.09×10^{-5}	3.86	9.5×10^{-3}	10.8	2^{20}

TABLE I. Parameters for our direct numerical simulations (DNSs): N is the number of collocation points, ν the kinematic viscosity, δt the time step, $L_{\rm I} \equiv \left(\sum_k |\hat{u}(k)|^2/k\right)/\left(\sum_k |\hat{u}(k)|^2\right)$ the integral length scale, $u_{\rm rms} \equiv \sum_k |\hat{u}(k)|^2$ the root-mean-square velocity, ${\rm Re} \equiv u_{\rm rms}L_{\rm I}/\nu$ the integral-scale Reynolds number, $\eta \equiv \left(\frac{\nu^3}{\epsilon}\right)^{1/4}$ the dissipation length scale, where ϵ is the energy dissipation rate, k_{max} the dealiasing cutoff, $T_L \equiv L_{\rm I}/u_{\rm rms}$ the large eddy turnover time, $\tau_\eta \equiv \left(\frac{\nu}{\epsilon}\right)^{1/2}$ the dissipation time scale, and N_p the number of tracers used in our Lagrangian studies.

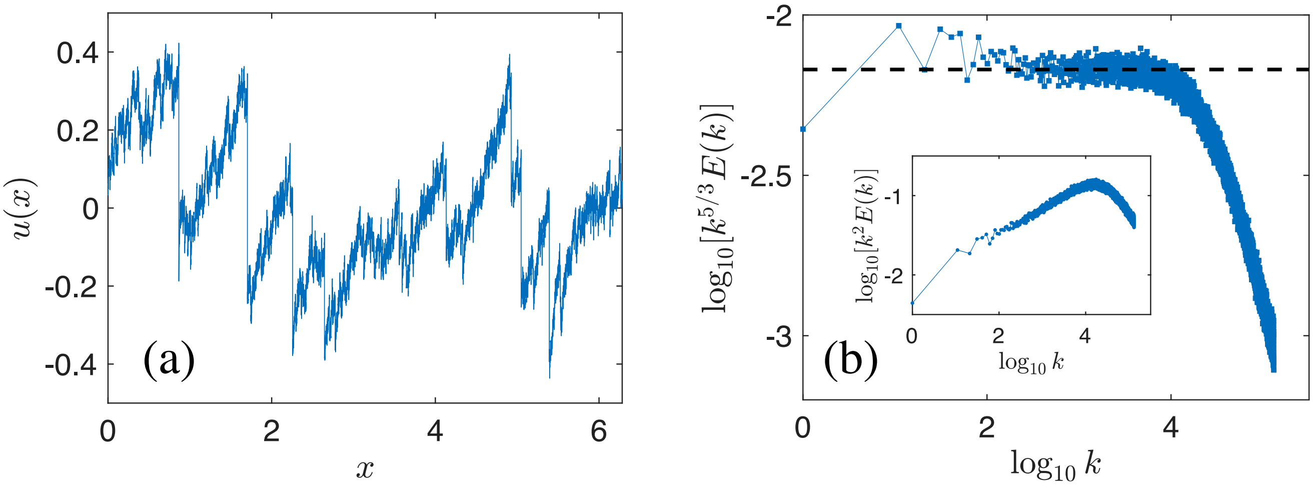

FIG. 1. (a) A plot versus x of the velocity profile $u(x)$ at a representative time in the non-equilibrium statistically steady state (NESS) of Eqs. (1) and (2) in the manuscript. (b) Log-log plot of the compensated energy spectrum, $k^{5/3}E(k)$ in this NESS, plotted up until the forcing-cutoff wave number $k_c = N/8$; the horizontal dashed line denotes the K41 scaling, $E(k) \sim k^{-5/3}$. *Inset:* Energy-dissipation spectrum $k^2E(k)$ plotted on a log-log scale up until $k_c = N/8$; the prominent peak in the graph indicates that the dissipation range is well-resolved. Both plots are obtained from run R2.

A representative velocity profile in the NESS and the compensated energy spectrum are shown in Fig. 1. The parameters for our DNSs are listed in Table I.

Quasi-Lagrangian (QL) velocity $V(x,t)$***:*** The QL velocities are calculated in the reference frame of a tracer which is advected by the local mean flow. In order to calculate the QL velocities, we load a single Lagrangian particle (tracer) at $x_0 = \pi$, i.e., in the middle of the simulation domain. At every iteration, we calculate the displacement $R(t)$ of the tracer, initially at the position x_0, by using the following equations:

$$\frac{dR(t)}{dt} = u(x_R,t)\,; \qquad x_R(t) = x_0 + R(t)\,; \tag{1}$$

$x_R(t)$ is the position of the tracer at time t and $u(x_R,t)$ is the Eulerian flow velocity at the location of the tracer. We solve the equation of motion for $R(t)$ by using the forward-Euler method. We then obtain the QL velocity

$$V(x,t) = u(x+R(t),t)\,. \tag{2}$$

For off-grid positions of a tracer, we need to find its velocity via interpolation. To circumvent interpolation errors, we calculate the components of the QL velocities exactly in Fourier space by using the relation $\hat{V}(k,t) = \hat{u}(k,\,t)e^{ikR(t)}$. After this we transform the QL velocities back to real space, such that $V(x,t) = \sum_k \hat{V}(k,t)e^{ikx} = \sum_k \hat{u}(k,t)e^{ik[x+R(t)]} = u(x+R(t),t)$.

II. PDF $\mathcal{P}(v_0)$ OF v_0

We assume that v_0 follows a standard normal distribution, i.e., $\mathcal{P}(v_0) \sim e^{-v_0^2/2}$, as in incompressible fluid turbulence. In order to justify our assumption, we calculate the complementary CPDF (c-CPDF), $Q_L(|v_0|)$, of the magnitude of v_0 by using the rank-order method. This circumvents binning errors which can affect the numerical determination of the PDF. In Fig. 2, we observe that the graph of $Q_L(|v_0|)$ is in good agreement with that of the c-CPDF of the standard normal distribution, thereby justifying our assumption. A similar result has been obtained earlier in Ref. [1].

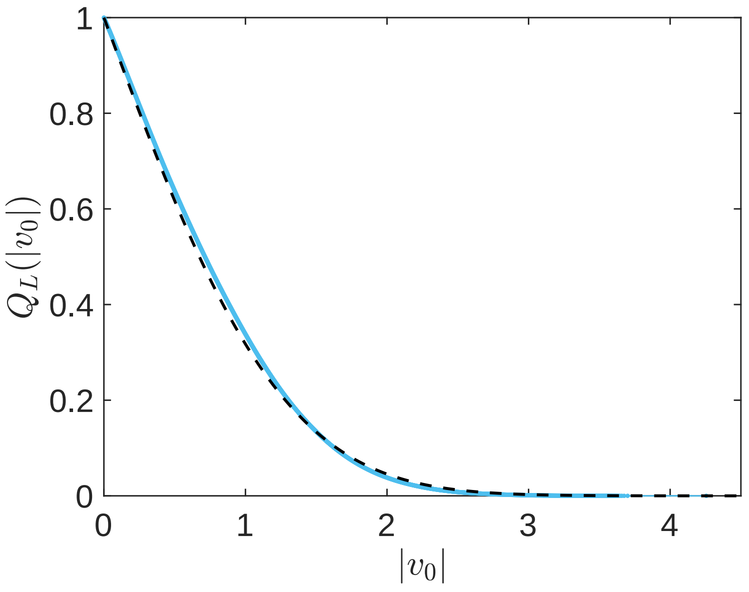

FIG. 2. Plot of the normalized c-CPDF $Q_L(|v_0|)$ of the magnitude of v_0 versus $|v_0|$, represented in blue; the black dashed curve is the c-CPDF of a standard normal distribution. The two curves match closely with each other, thereby justifying our assumption in the main text.

III. SCALING OF THE MOMENTS OF $\tau_{\rm col}$ WITH r

The order-p moment of τ is

$$T^{\rm p}_{\rm col}(r) = \langle \tau^p \rangle = \int_0^\infty \tau^p \Phi(\tau) d\tau \,. \tag{3}$$

$T^{\rm p}_{\rm col}$ has contributions from three different terms on the RHS of Eq. (23) in the manuscript, so we analyze it term by term.

(A) The contribution to $T^{\rm p}_{\rm col}$ from the first term on the RHS of Eq. (23) in the manuscript is

$$T^{\rm p}_{{\rm col},A}(r) \sim r^{2/3} \int_0^\infty \tau^{p-2} \exp\left[-\frac{r^{4/3}}{2\tau^2}\right] d\tau \,. \tag{4}$$

By making the substitution $s = \sqrt{2}\tau/r^{2/3}$, we get

$$T^{\rm p}_{{\rm col},A}(r) \sim r^{2p/3} \int_0^\infty s^{p-2} \exp\left[-\frac{1}{s^2}\right] ds \,, \tag{5}$$

which implies,

$$T^{\rm p}_{{\rm col},A}(r) \sim r^{2p/3} \,. \tag{6}$$

(B1) The contribution from the second term on the RHS of Eq. (**??**) is

$$T^{\rm p}_{{\rm col},B1}(r) \sim r^2 \int_0^\infty \tau^{p-2} \exp\left[-\frac{r^2}{2\tau^2}\right] d\tau\,. \tag{7}$$

By making the substitution $s=\sqrt{2}\tau/r$, we get

$$T^{\rm p}_{{\rm col},B1}(r) \sim r^{p+1} \int_0^\infty s^{p-2} \exp\left[-\frac{1}{s^2}\right] ds\,. \tag{8}$$

This implies that

$$T^{\rm p}_{{\rm col},B1}(r) \sim r^{p+1}\,. \tag{9}$$

(B2) The contribution from $\Phi_{B2}(\tau)$ is,

$$T^{\rm p}_{{\rm col},B2}(r) \sim \sum_{t_*}\left[W_{t_*} r \int_{t_*}^\infty \frac{\tau^p}{(\tau-t_*)^2}\exp\left\{-\frac{r^2}{2(\tau-t_*)^2}\right\} d\tau\right]\,, \tag{10}$$

where $W_{t_*}=w(t_*)$. By making the substitution $s=r/(\tau-t_*)$, we get

$$T^{\rm p}_{{\rm col},B2}(r) \sim \sum_{t_*}\left[W_{t_*}\int_0^\infty \left(\frac{r}{s}+t_*\right)^p e^{-s^2/2} ds\right]\,. \tag{11}$$

We now expand $\left(\frac{r}{s}+t_*\right)^p$ in a binomial series. In order to ensure its convergence for the different values of s, given the values of t_* and r, we write

$$\left(t_*+\frac{r}{s}\right)^p = \begin{cases} t_*^p\left(1+\frac{r}{st_*}\right)^p & \text{if} \quad s>\frac{r}{t_*}\,; \\ \frac{r^p}{s^p}\left(1+\frac{st_*}{r}\right)^p & \text{if} \quad s<\frac{r}{t_*}\,. \end{cases} \tag{12}$$

Then, Eq. (11) can be written as

$$T^{\rm p}_{{\rm col},B2}(r) \sim \sum_{t_*}\left[W_{t_*}\left(I_1+I_2\right)\right]\,,$$

where

$$I_1 = r^p\int_0^{r/t_*}\frac{1}{s^p}\left(1+\frac{st_*}{r}\right)^p e^{-s^2/2}ds = r^p\int_0^{r/t_*}\left[s^{-p}+\frac{pt_*}{r}s^{1-p}+p(p-1)\left(\frac{t_*}{r}\right)^2 s^{2-p}+...\right]e^{-s^2/2}ds \tag{13}$$

and

$$I_2 = t_*^p\int_{r/t_*}^\infty\left(1+\frac{r}{st_*}\right)^p e^{-s^2/2}ds = t_*^p\int_{r/t_*}^\infty\left[1+p\frac{r}{st_*}+p(p-1)\left(\frac{r}{st_*}\right)^2+...\right]e^{-s^2/2}ds\,. \tag{14}$$

By making the substitution $y=s^2/2$, the integrals over each term in Eq. (13) can be be written in terms of the lower incomplete gamma function $\gamma(n,x)=\int_0^x t^{n-1}e^{-t}dt$. We use the asymptotic form of $\gamma(n,x)$, in the limit of $x\to 0$, $\gamma(n,x)\sim\frac{x^n}{n}$, because $r\ll 1$. We find that the leading-order contribution from I_1 scales as r^2.

By making the same substitution in Eq. (14), we find that each one of the integrals in Eq. (14) can be written in terms of the upper incomplete gamma function $\Gamma(n,x)=\int_x^\infty t^{n-1}e^{-t}dt$. By using the asymptotic properties of the resulting terms in the limit of $r\ll 1$, we find that the $r-$dependent leading-order contribution scales linearly with r, with logarithmic corrections. As a result,

$$T^{\rm p}_{{\rm col},B2}(r) \sim C_1 r + C_2 r\ln r\,, \tag{15}$$

where $C_1\sim\sum_{t_*}\left[\frac{p}{2t_*}(\gamma-2\ln t_*)\right]$ and $C_2\sim\sum_{t_*}\left(\frac{p}{t_*}\right)$ and γ the Euler constant. Given the scaling forms of τ in Eqs. (8), (10) and (14) in the manuscript, and $T_L\sim L/v_0$, $\tau\equiv\tau_{\rm col}(\ell)/T_L$, $r\equiv\ell/L$, $\tau\ll 1$ for all collapsing intervals (recall that $t_*<\tau$), $C_1\gg C_2$ for all $p>0$, whence we have

$$T^{\rm p}_{{\rm col},B2}(r) \sim r\,. \tag{16}$$

By combining Eqs. (9), (6) and (16), we obtain:

$$T^{\rm p}_{\rm col}(r) \sim \begin{cases} r^{2p/3} & \text{for} \quad p\le 3/2\,; \\ r & \text{for} \quad p>3/2\,. \end{cases} \tag{17}$$

In this way, we are able to extract from $P(\tau_{\rm col})$ the same scaling behavior of the moments of $\tau_{\rm col}$ as we had obtained from our theoretical arguments given in the main paper.

IV. EQUAL-TIME STRUCTURE FUNCTIONS

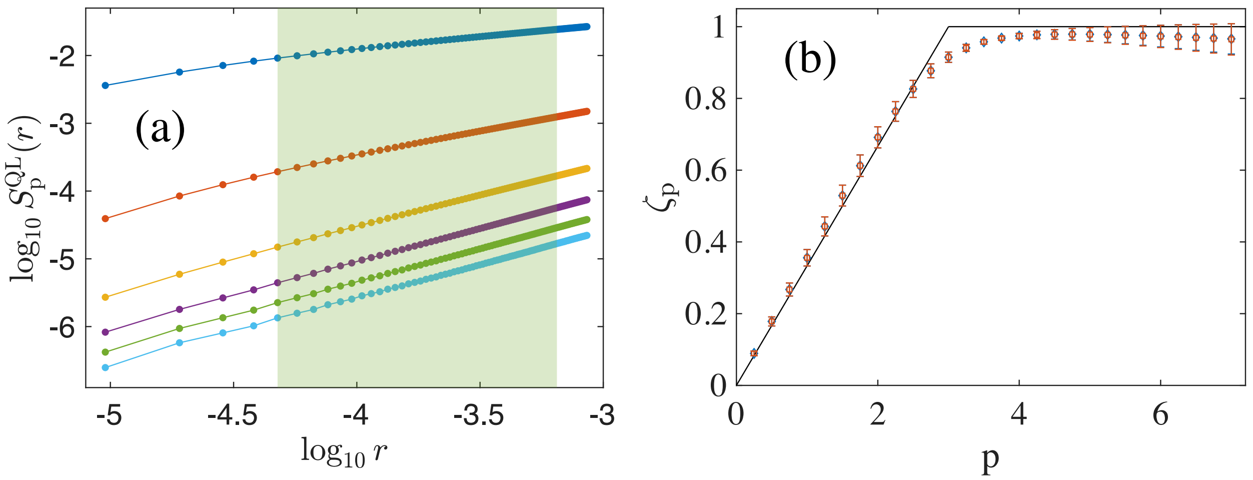

FIG. 3. Log-log plots of QL equal-time structure functions $S_p^{QL}(r)$ for $p = 1$ (dark blue), $p = 2$ (red), $p = 3$ (yellow), $p = 4$ (violet), $p = 5$ (green) and $p = 6$ (light blue); the green shaded portion indicates the range over which we carry out local-slope analysis for calculating ζ_p^{QL}. (b) Equal-time Eulerian (blue diamonds) and QL (red squares) structure function exponents, ζ_p^{Eu} and ζ_p^{QL}, for different values of p; the black lines denote the bifractal exponents given by Eq. (5)c in the manuscript.

V. DERIVATION OF THE COLLAPSE-TIME EXPONENTS FROM THE MULTIFRACTAL MODEL OF TURBULENCE

In the multifractal model of turbulence, the probability of being within a spatial distance r of a set of fractal dimension $D(h)$ scales as $r^{d-D(h)}$, where d is the embedding dimension of the velocity field. This is the same as the probability, p_r, of finding a Lagrangian interval of length r across a set of fractal dimension $D(h)$. By using $\tau_{col} \sim r/\delta u(r) \sim r^{1-h}$, we write

$$T_{col}^{p}(r) \sim \int_h r^{p(1-h)+1-D(h)} d\mu(h) \sim r^p \int_h r^{-ph+1-D(h)} d\mu(h) \sim r^{z_{col}^{p}}\,, \tag{18}$$

where $\mu(h)$ is the weight associated with each h and $h \in [h_{min}, h_{max}]$. From the definition of equal-time structure functions $S_p(r)$ and by using the multifractal model we have

$$S_p(r) \sim \int_h r^{ph+1-D(h)} d\mu(h) \sim r^{\zeta_p}\,, \tag{19}$$

whence we obtain the following bridge relation between z_{col}^{p} and ζ_p:

$$z_{col}^{p} = p + \zeta_{-p}\,. \tag{20}$$

By assuming that $S_p(r) \equiv \langle|\delta u(r)|^p\rangle$ exists for $p < 0$, we have $\zeta_p = p/3$ for all $p < 0$. Hence, we get the following bridge relation for z_{col}^{p}:

$$z_{col}^{p} = \frac{2p}{3}, \quad \text{for all p.} \tag{21}$$

This agrees with the one derived earlier from the tracer dynamics [Eq. (17b) in the manuscript] up to $p = 3/2$. As we saw before, the major contribution to z_{col}^{p} for $0 \le p \le 3/2$ mainly comes from those collapsing intervals whose dynamic properties are not significantly affected by shocks. The saturation of z_{col}^{p} to unity for $p > 3/2$ occurs because of the change in the scaling of the velocity fluctuations across a collapsing interval because of the appearance of a shock at $t \equiv t_* \ll \tau_{col}$. This cannot be explained, simply, by the multifractal model alone.

---

* sadhitrode@iisc.ac.in
† dhruba.mitra@gmail.com
‡ rahul@iisc.ac.in

[1] S. Boldyrev, T. Linde, and A. Polyakov, Velocity and Velocity-Difference Distributions in Burgers Turbulence, Phys. Rev. Lett. **93**, 184503 (2004).
